# Intention Detection Strategies for Robotic Upper-Limb Orthoses: A Scoping Review Considering Usability, Daily Life Application, and User Evaluation

**DOI:** 10.3389/fnbot.2022.815693

**Published:** 2022-02-21

**Authors:** Jessica Gantenbein, Jan Dittli, Jan Thomas Meyer, Roger Gassert, Olivier Lambercy

**Affiliations:** ^1^Rehabilitation Engineering Laboratory, Department of Health Sciences and Technology, ETH Zurich, Zurich, Switzerland; ^2^Future Health Technologies, Singapore-ETH Centre, Campus for Research Excellence and Technological Enterprise (CREATE), Singapore, Singapore

**Keywords:** intention detection, wearable robotics, upper limb orthosis, user studies, human robot interaction, usability evaluation

## Abstract

Wearable robotic upper limb orthoses (ULO) are promising tools to assist or enhance the upper-limb function of their users. While the functionality of these devices has continuously increased, the robust and reliable detection of the user's intention to control the available degrees of freedom remains a major challenge and a barrier for acceptance. As the information interface between device and user, the intention detection strategy (IDS) has a crucial impact on the usability of the overall device. Yet, this aspect and the impact it has on the device usability is only rarely evaluated with respect to the context of use of ULO. A scoping literature review was conducted to identify non-invasive IDS applied to ULO that have been evaluated with human participants, with a specific focus on evaluation methods and findings related to functionality and usability and their appropriateness for specific contexts of use in daily life. A total of 93 studies were identified, describing 29 different IDS that are summarized and classified according to a four-level classification scheme. The predominant user input signal associated with the described IDS was electromyography (35.6%), followed by manual triggers such as buttons, touchscreens or joysticks (16.7%), as well as isometric force generated by residual movement in upper-limb segments (15.1%). We identify and discuss the strengths and weaknesses of IDS with respect to specific contexts of use and highlight a trade-off between performance and complexity in selecting an optimal IDS. Investigating evaluation practices to study the usability of IDS, the included studies revealed that, primarily, objective and quantitative usability attributes related to effectiveness or efficiency were assessed. Further, it underlined the lack of a systematic way to determine whether the usability of an IDS is sufficiently high to be appropriate for use in daily life applications. This work highlights the importance of a user- and application-specific selection and evaluation of non-invasive IDS for ULO. For technology developers in the field, it further provides recommendations on the selection process of IDS as well as to the design of corresponding evaluation protocols.

## 1. Introduction

Our upper limbs are essential for numerous tasks in our daily lives, allowing us to interact physically and socially with our environment. Functional limitations of the upper limbs, e.g., due to impairment from neurological injury or disease, may have a substantive impact on independence, health, and wellbeing of the people affected, not only on physical but also on emotional, cognitive, and behavioral levels (Poltawski et al., 2016). In recent years, robotic wearable orthoses for the upper limbs, i.e., for the shoulder, elbow, wrist, hand or fingers, emerged as tools to compensate for functional impairments and therefore aim to improve quality of life of their users. Orthoses assist movements by being worn around and operated in parallel to the user's impaired limb (Tucker et al., 2015). The potential of wearable upper-limb orthoses (ULO), in this context often called “exoskeletons,” has further been exploited not only for people with impairments but also to complement or augment upper limb function of non-impaired users, e.g., by enhancing their strength or endurance in specific tasks in their work environment (Bergamasco and Herr, 2016; Thalman and Artemiadis, 2020).

However, wearable robotic orthoses are not yet easily available and widely accepted by end-users. Previous studies have shown that insufficient usability can lead to low user acceptance of assistive technologies such as ULO, and consequently to high device abandonment rates (Biddiss and Chau, 2007; Ravneberg, 2012; Sugawara et al., 2018). The usability of a device describes how well it can be used by a specific user and context of use (ISO 9241-11, 2018). A critical factor in the use of an ULO is the way the user can trigger the desired robot motion. Thus, we hypothesize that the intention detection strategy (IDS) as the interface between users and their ULO plays an essential role in the usability of the overall device perceived by the user. Therefore, ensuring a high usability of an IDS is crucial to promote the adoption of an ULO to its targeted context of use. The question of whether a person is able and willing to use a specific IDS also highly depends on the person's residual sensorimotor capabilities and the tasks for which the device is intended. As such, the appropriateness of an IDS for an ULO depends not only on its technical advantages and limitations but also on the target user and the intended usage scenario. However, research papers describing the development or application of IDS for ULO rarely cover all these decisive factors.

Previous reviews have provided exhaustive overviews of IDS for movement assistive devices. Lobo-Prat et al. (2014) reviewed non-invasive IDS for active movement assistive devices in general, not specifically focusing on ULO. Other reviews were published focusing on specific strategies based on electromyography (EMG) (Parajuli et al., 2019; Hameed et al., 2020; Rodríguez-Tapia et al., 2020) or brain-computer-interfaces (BCI) (Millán et al., 2010; Khan et al., 2020; Rashid et al., 2020). However, the scopes of these reviews only cover a specific section of the broad range of available IDS. Chu and Patterson (2018) and du Plessis et al. (2021) published narrative reviews discussing robotic devices for hand rehabilitation and assistance, in which also IDS were briefly discussed. However, all these existing reviews primarily focus on the concepts and technical design of IDS. As such, a review systematically analyzing various IDS with respect to their daily life applicability and usability is yet missing.

Through this work, we aim to provide technology developers with an evidence-based overview of the key aspects to consider for the selection of appropriate non-invasive IDS for ULO applications. We further present recommendations for the choice of usability attributes to promote a more comprehensive and standardized evaluation of IDS for ULO from a user-centered point of view. This work is important, as it provides a novel analysis of how different IDS are applied and in which context, paving the way for more informed selection of IDS in wearable robotics, which could ultimately improve the acceptance of such technologies.

## 2. Methods

This scoping review was conducted in compliance with the Preferred Reporting Items for Systematic Reviews and Meta-Analyses requirements extension for scoping reviews (PRISMA-ScR) (Tricco et al., 2018).

### 2.1. Literature Search

**Figure 1:**
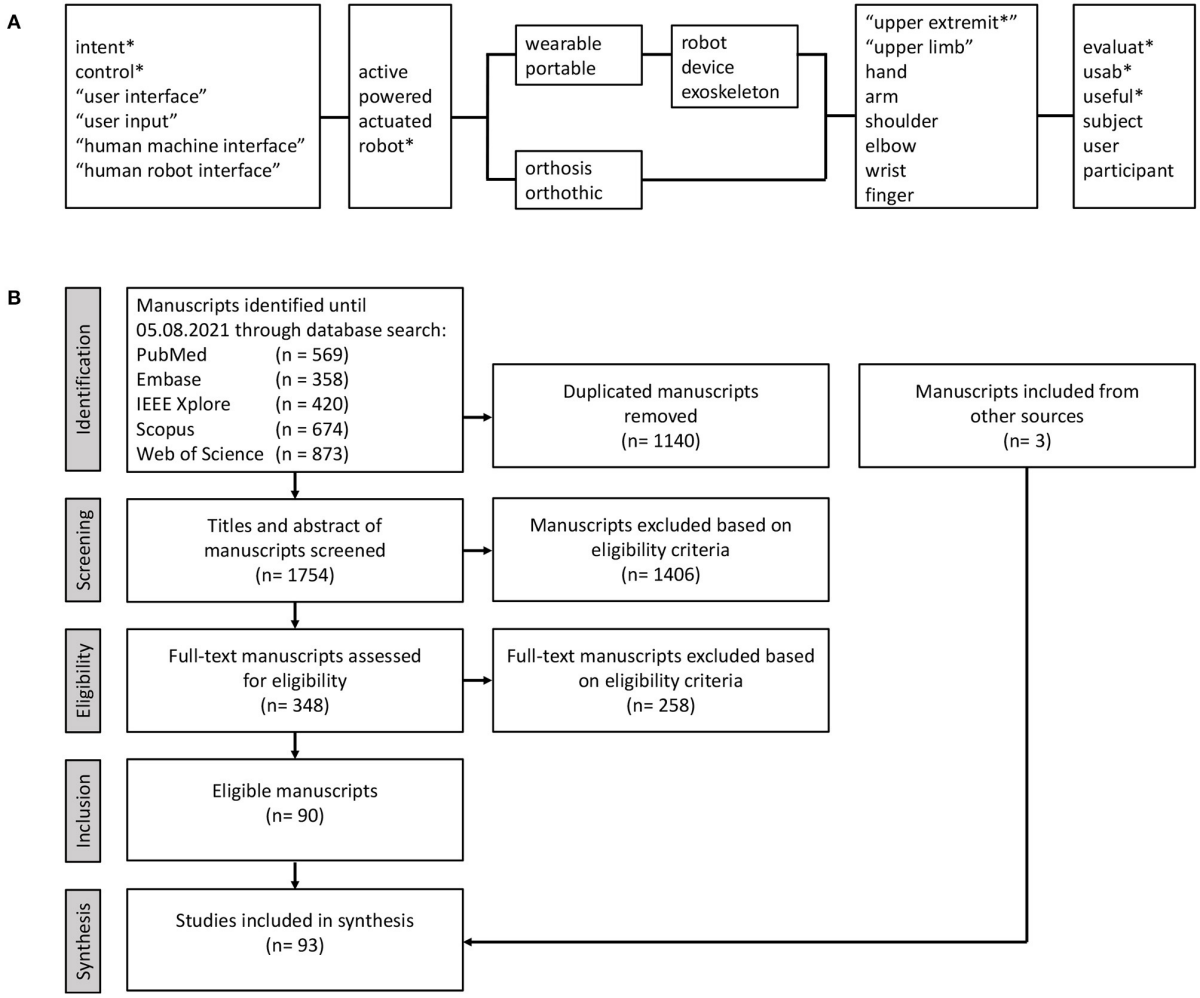
Methods of manuscript search and study selection. **(A)** Exemplary search string used for Web of Science. Parallel blocks denote OR-operator, serial blocks denote AND-operator, asterisk (*) denotes truncation-operator. **(B)** PRISMA-ScR flowchart for the conducted literature search. Flowchart adapted from Moher et al. (2009).

A literature search was conducted in August 2021 on five electronic databases (Web of Science, Scopus, PubMed, Embase, and IEEE Xplore). An example of the search string used for Web of Science is shown in Figure 1A. Search strings for the other databases were built analogously but adapted to the databases' specific requirements wherever needed.

### 2.2. Study Selection

In order to select eligible studies from the obtained manuscripts, a set of criteria were predefined.

Inclusion criteria were: (I1) manuscripts describing the evaluation of a human intent-controlled wearable or portable ULO or non-invasive IDS used in combination with an ULO; (I2) full-text manuscripts in English language.

Exclusion criteria were: (E1) manuscripts not providing information on which IDS was used; (E2) IDS requiring invasive interventions; (E3) evaluation not involving human participants wearing the ULO during data collection; (E4) non-real-time control of the ULO; (E5) third person, autonomous, purely gravity-compensating, or master-slave controlled ULO (i.e., not based on intent from the user); (E6) studies only considering rehabilitative effects of the ULO over multiple sessions as outcome; (E7) same IDS and ULO already assessed in a newer included study by same authors.

The reasons for choosing these exclusion criteria were either because the focus of the considered manuscript was not within the review targeting user evaluation of IDS (E1, E2, E5, E6), because the methods of the study did not sufficiently reflect the actual intended use of the ULO in daily life, i.e., the validity of the user evaluation results for application in daily life was not given (E3, E4), or because the core information of the study was duplicated, e.g., pilot study and subsequent full study (E7). In case a manuscript discussed an evaluation protocol consisting of multiple methods, of which not all comply with the exclusion criteria (e.g., offline and online assessment of reliability within the same manuscript), the manuscript was included, but only the methods complying with the defined criteria were considered for the data extraction. In case a manuscript did not provide sufficient information to determine its compliance with a specific exclusion criterion, the manuscript was included, provided that it complied with all the other criteria. Since this scoping review aims to provide an overview of all the studies evaluating ULO controlled by an IDS, no critical appraisal was conducted and thus no studies were excluded based on their methodological quality.

Screening of the manuscripts based on these eligibility criteria was conducted by three unblinded reviewers (JG, JD, JTM) using the online systematic review management tool Covidence (Veritas Health Innovation Ltd., Australia). For title and abstract screening, the first half of the manuscripts was screened by two reviewers independently, and a third reviewer was consulted in case of disagreements. Single screening of the second half of manuscripts and subsequent full texts screening was split between the three reviewers, and a second reviewer was consulted in case of uncertainty.

### 2.3. Data Extraction

A data extraction form was developed in a spreadsheet software (Microsoft Excel, Microsoft Corporation, USA) and piloted by two reviewers (JG, JD). Subsequent data extraction was conducted in Excel by the leading reviewer (JG) to ensure consistency.

The core part of the data extraction consisted of usability evaluation findings reported in the studies, which were structured according to a list of 12 usability attributes. The international standard ISO 9241-11 defines usability as “*the extent to which a system, product, or service can be used by specified users to achieve specified goals with effectiveness, efficiency, and satisfaction in a specified context of use”* (ISO 9241-11, 2018), whereas in this work, we considered IDS as the “*system, product, or service”* of interest. The 12 usability attributes presumed to be relevant for IDS were selected based on the findings of a recent survey on common practices in usability evaluation of wearable robots (Meyer et al., 2021). For the purpose of grouping information within this review, each usability attribute was also assigned to one of three usability dimensions: “effectiveness” defined as “*the accuracy and completeness with which users achieve specified goals”*, “efficiency” as “*the resources set in relation to the results achieved,”* and “satisfaction” as “*the extent to which the user's physical, cognitive, and emotional responses that result from the use of a system, product, or service meet the user's needs and expectations”* (ISO 9241-11, 2018). The list of usability attributes and the corresponding usability dimensions is shown in Table 1. General usability assessments which could not be assigned to a specific attribute were not included in the data extraction since they do not provide sufficiently detailed information to be synthesized.

**Table 1:** Predefined list of usability attributes and their definitions applied in regards to IDS.

**Group**	**Attribute**	**Definition applied**
Effectiveness	Reliability	Does the IDS perform its requested functions under stated conditions?
	Robustness	Does the IDS continue to function in the presence of invalid inputs or stressful or changing environmental conditions?
Efficiency	Mental workload	How mentally demanding is the generation of a command?
	Physical workload	How physically demanding is the generation of a command?
	Temporal workload	How much time does the generation of a command take (incl. computational time, excl. practice and classifier training)?
	Learnability	What influence does practice have on the ability to generate a command?
	Ease-of-use	How easy does the user find the generation of a command?
	Cost	What are acquisition and/or maintenance costs (financial or time)?
Satisfaction	Naturalness	How natural does the generation of a command feel to the user compared to unimpaired movement?
	Comfort	How physically comfortable and ergonomic does the user perceive the IDS during use?
	Simplicity of setup	How simple is the setup of the IDS (e.g., to calibrate, or to don & doff)?
	Enjoyability	How much did the user enjoy using the IDS (e.g., in terms of mood, motivation, frustration)?

Besides the usability attributes, the data extraction form included technical information to provide an overview of existing IDS and information about the context of use, i.e., the ULO for which the IDS was used, the target user, and the intended application. Lastly, information about the user evaluation methods used in the studies was extracted, including information about the used test protocol and the number and kind of participants.

### 2.4. Data Synthesis

#### 2.4.1. Synthesis of Assessed Usability Attributes

Depending on the aim of a study, only a subset of usability attributes might be considered relevant by technology developers and was thus included in the study protocol. In order to investigate which usability attributes were assessed the most, the number of studies in which a particular usability attribute was evaluated was determined. An attribute was counted as “assessed,” if it was evaluated or discussed quantitatively or qualitatively in a study. Depending on whether a study assessed the usability attribute directly based on the defined user evaluation protocol or indirectly based on the observation or interpretation of the study authors, the collected data was categorized as “data-driven finding” or “indirect finding,” respectively. The methods with which each attribute was assessed were summarized and will be described in Section 3.2.

#### 2.4.2. Synthesis of Types of IDS

To classify the IDS, the four-level classification scheme for IDS proposed by Lobo-Prat et al. (2014) was adapted. Following the user-centered scope of this review, we considered the user's body part that generates the command, i.e., the local source of the input signal, as level 1. This source directly relates to the user's residual functional capabilities and thus their ability to use a specific IDS. On level 1, the IDS were assigned to three groups. “IDS sourcing from targeted upper-limb segment(s)” were defined as those related to the physiological execution of the desired movement, e.g., actuation of the finger joints of the ULO initiated by residual movement of the fingers or activation of the finger muscles in the ipsilateral forearm. On the contrary, “IDS sourcing from non-targeted upper-limb segments” were defined as those not relating to the physiological execution of the desired movement but still sourcing from the upper limb. It should be noted that the “non-targeted upper-limb segment(s)” does not necessarily imply that the source is contralateral to the upper limb targeted by the ULO. For example, actuation of the finger joints of the ULO could be initiated by muscle activation from the ipsilateral upper-arm or from the forearm muscles contralateral to the ULO. “IDS sourcing from other body parts” were defined as all remaining IDS, e.g., sourcing from the brain or the tongue. Levels 2–4 of the classification scheme were defined similarly to Lobo-Prat et al. (2014) as: the corresponding physiological phenomena related to the IDS used (level 2), the corresponding measured signals (level 3), and the used sensors to measure these signals (level 4).

When analyzing the findings from the usability evaluations (Section 3.3), the IDS are discussed on level 2, since the most distinct differences in perceived usability were expected on that level. In the following, each IDS from level 2 are described in three parts: first, by a general introduction of the principle and overall strengths and benefits, second by reviewing studies which used the described IDS, and third, by synthesizing results with respect to usability attribute data extracted from the included studies. For the IDS where no usability attribute data was reported in the studies, this is stated accordingly. Due to the heterogeneity and partially qualitative nature of this data, the results of the synthesis will be described narratively, and IDS are compared to each other only on a qualitative level where possible.

## 3. Review

### 3.1. Characteristics of Included Studies

After the full-text screening of 348 manuscripts, 93 eligible studies were identified and included in the data extraction. The flowchart of study inclusion is given in Figure 1B. Included studies were published between 2000 and 2021, whereas 50.5% were published since 2018. A condensed version of the data extraction tables, including references to all included studies, can be found in Supplementary Material 1. The complete, detailed data extraction table can be obtained from the authors upon request.

**Figure 2:**
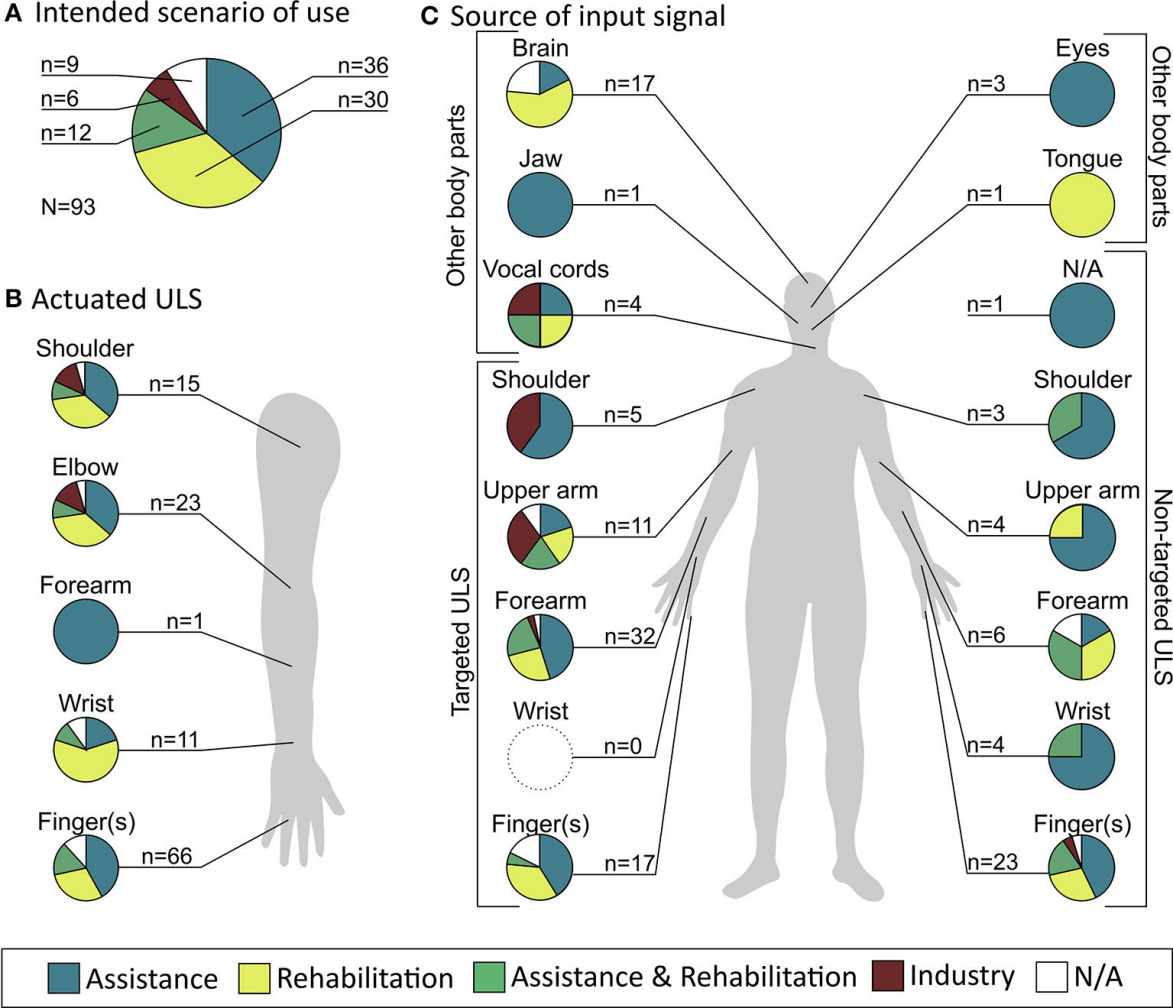
Overview of characteristics of upper limb orthoses (ULO) assessed and sources of input signals. **(A)** Distribution of contexts of use of ULO over all included studies. **(B)** Distribution of contexts of use in relation to the upper limb segment (ULS) actuated by the ULO. **(C)** Distribution of contexts of use of ULO in relation to the source of input signal.

An overview of the distribution of the intended scenarios of use of the ULO, as well as the actuated upper-limb segments by these ULO, is given in Figures 2A,B, respectively. 83.9% of the ULO were intended to be used as assistive and/or rehabilitative device, whereas the occurrences of these two groups were relatively well balanced. The actuated joints or movements of these ULOs were finger joint(s) (70.9%), elbow joint (24.7%), shoulder joint (16.1%), wrist joint (11.8%), and forearm pronation/supination (1.1%). Figure 2C summarizes which scenarios of use were intended for which IDS sourcing from specific body parts (i.e., classification level 1). Assistive applications were targeted in 56.9% of ULO with IDS sourcing from targeted upper-limb segments and in 70.7% of ULO with IDS sourcing from non-targeted upper-limb segments. Rehabilitative applications were targeted in 43.1 and 41.5%, respectively of the same groups. For IDS sourcing from other body parts, those from brain signals were more often targeting rehabilitative applications (58.8%) while those sourcing from eyes, jaw, tongue, and vocal cords were more often targeting assistive applications (62.5%). Most industrial applications were targeted for ULO with IDS sourcing from targeted upper-limb segments (75%).

A distinctive eligibility criterion of this scoping review was the exclusion of studies that did not involve any human participants in the evaluation of the ULO controlled by the specific IDS. On average, 5.044.41 (SD 4.41) participants were involved in the studies, of which 2.07 (SD 3.42) participants belonged to the stated target population. Overall, at least one target user was involved in 40.9% of studies, whereas 46.2% involved only participants not belonging to the target population, and 12.9% did not provide sufficient information to determine whether the participants belonged to the target population.

A total of 28 different IDS were disclosed in the included studies and were organized in Table 2 according to the proposed four-level classification scheme. Of the 93 included studies, 69 used IDS with sources from the targeted upper-limb segments, 40 used IDS with sources from non-targeted upper-limb segments, and 26 used IDS with sources from other body parts. The total number of 133 exceeds the number of included studies (*n* = 93) as some studies reported more than one and up to four IDS. A total of 14 studies assessed ULOs with multimodal IDS, i.e., multiple different IDS used simultaneously, whereas 20 studies assessed ULOs, which allowed to choose between different IDS and compare performance.

**Table 2:** Classification of intention detection strategies.

			**Level 1: Source of input signal**																		
			**Targeted ULS**					**Non-targeted ULS**						**Other body parts**							
**Level 2: Physiological phenomenon**	**Level 3: Signal**	**Level 4: Sensor**	**Finger**	**Wrist**	**Forearm**	**Upper arm**	**Shoulder**	**Not defined**	**Finger**	**Wrist**	**Forearm**	**Upper arm**	**Shoulder**	**Jaw**	**Tongue**	**Vocal cords**	**Eyes**	**Brain**	**Total level 4**	**Total level 3**	**Total level 2**
Muscle activation	EMG	Electrodes	0	0	22	8	5	1	0	0	5	4	2	0	x	x	x	x	47	47	47
Muscle contraction	FMG	Force sensors	0	0	3	1	0	0	0	0	1	0	0	1	x	x	x	x	6	6	6
Isometric force	Exerted force/torque	Force/torque sensors	13	0	6	0	0	0	1	0	0	0	0	0	x	x	x	x	20	20	20
		IMUs	0	0	1	2	0	0	0	1	0	0	0	x	x	x	x	x	4		
	Kinematics	Load cells	0	0	0	0	0	0	0	0	0	0	1	x	x	x	x	x	1	5	
UL movement	Joint rotation	Bending sensors	4	0	x	x	0	0	0	3	x	x	0	x	x	x	x	x	7	7	12
Tongue movement	Magnetic field	Magnet sensor	x	x	x	x	x	x	x	x	x	x	x	x	1	x	x	x	1	1	1
	EOG	Electrodes	x	x	x	x	x	x	x	x	x	x	x	x	x	x	2	x	2	2	
Eye movement	Corneal reflection	Cameras	x	x	x	x	x	x	x	x	x	x	x	x	x	x	1	x	1	1	3
	EEG	Electrodes	x	x	x	x	x	x	x	x	x	x	x	x	x	x	x	16	16	16	
Brain activity	fNIRS	Optodes	x	x	x	x	x	x	x	x	x	x	x	x	x	x	x	1	1	1	17
Speech	Sound	Microphones	x	x	x	x	x	x	x	x	x	x	x	x	x	4	x	x	4	4	4
		Buttons/switches	x	x	x	x	x	x	15	x	x	x	x	x	x	x	x	x	15		
		Joysticks	x	x	x	x	x	x	4	x	x	x	x	x	x	x	x	x	4		
N/A	Manual trigger	Touchscreens	x	x	x	x	x	x	3	x	x	x	x	x	x	x	x	x	3	22	22
			17	0	32	11	5	1	23	4	6	4	3	1	1	4	3	17			
		**Total level 1**	65					41						26					**132**		

*The four-level classification scheme was adapted from Lobo-Prat et al. (2014). Columns show the first level (source of input signal), rows show second to fourth levels (physiological phenomenon, signal, sensor). The numbers indicate the number of included studies, which used the corresponding IDS, (x) indicates technically impossible IDS. Total number of included studies per IDS for each level is indicated. ULS, upper limb segment; EMG, electromyography; FMG, force myography; EOG, electrooculography; EEG, electroencephalography; N/A, not applicable. Shades of grey from dark to bright represent four classification levels from 1 to 4*.

### 3.2. Usability Attributes and Methods of Evaluation

**Figure 3:**
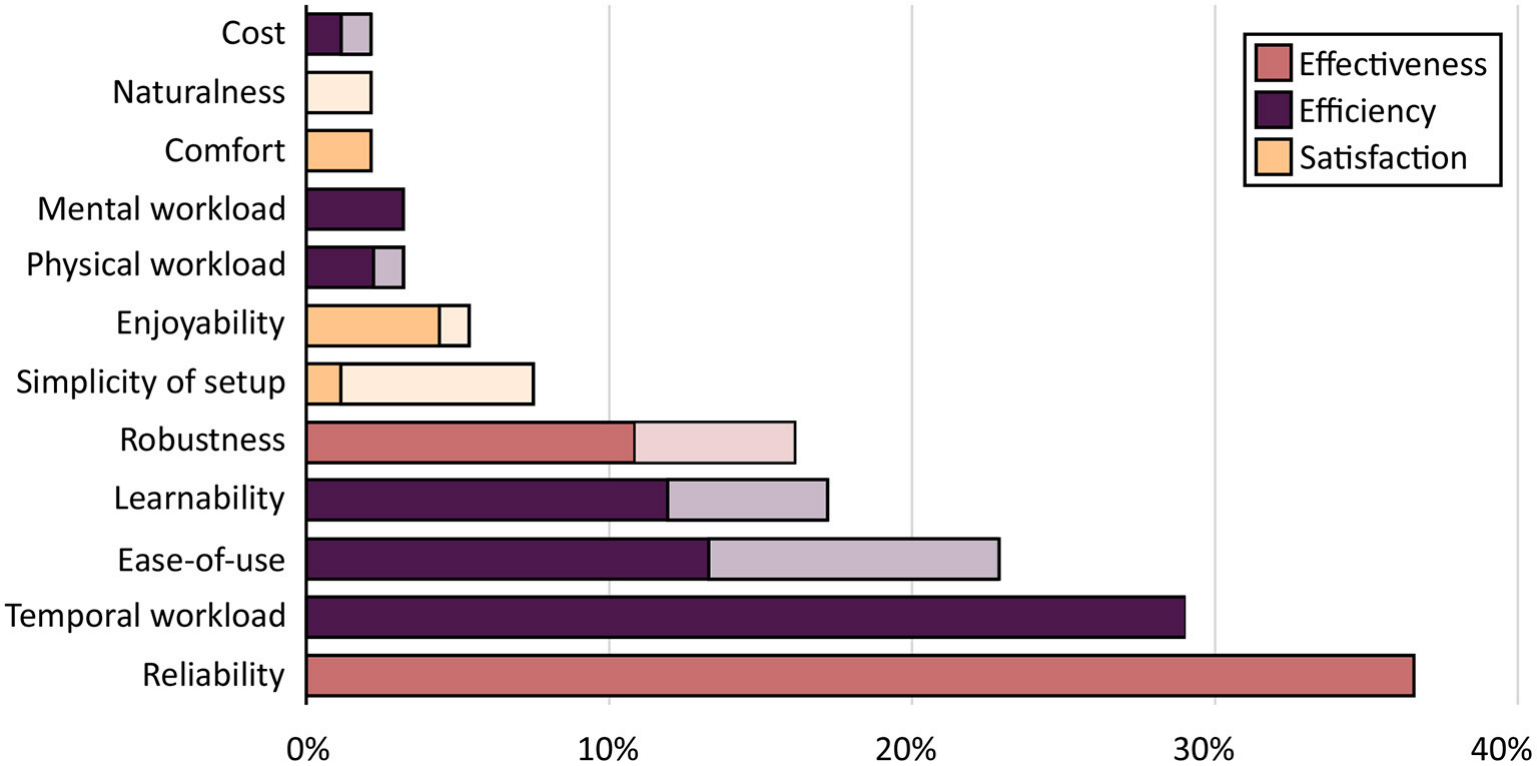
Frequency of assessment of usability attributes. List of usability attributes ranked by the percentage of studies, in which they were assessed. Colors indicate the assigned usability grouping. Dark bar sections indicate “data-driven findings,” bright bar sections indicate “indirect findings.”

Out of the twelve usability attributes defined in Table 1, the studies assessed on average 1.47 (SD 1.50) different attributes related to the IDS, whereas 31 studies did not assess a single attribute specifically related to the IDS. From all reported assessments of usability attributes, 75.5% were direct data-driven findings. The three attributes related to the IDS which were assessed the most were “reliability” (36.6%), “temporal workload” (29.0%), and “ease of use” (22.6%). “Cost,” “naturalness,” and “comfort” were the least assessed attributes being reported in only two studies each. Usability attributes assigned to the usability dimensions “effectiveness” and “efficiency” appeared to be more frequently assessed, compared to attributes assigned to “satisfaction.” The frequency of assessment of usability attributes along with the distribution of direct data-driven findings and indirect statements is shown in Figure 3. The methods of assessing individual usability attributes are further discussed below, in order of decreasing frequency of occurrence in the included studies.

#### 3.2.1. Reliability

Reliability was the most frequently assessed attribute, with all data-driven findings being reported in 35 studies. In the context of IDS, reliability is alternatively also often called accuracy and describes how good an IDS performs its requested functions under stated, non-varying conditions. However, in real-time control, the stated, non-varying conditions are often difficult to adhere to, making a clear differentiation between reliability and robustness difficult.

Some studies did report reliability qualitatively based on user feedback (e.g., Song and Chai, 2013). However, most of the included studies did express it as the number or percentage of successes or errors (e.g., Park et al., 2019) or classification accuracy of the IDS (e.g., Lu et al., 2019). Some subdivided these classes further according to the type of success or error (e.g., Zhou et al., 2019), i.e., true/false positives or true/false negatives. Many studies compared their achieved success or error rates to those of other studies to rate their IDS. However, no studies reported a generally accepted target value a good IDS should achieve in terms of reliability.

#### 3.2.2. Temporal Workload

The temporal workload was assessed in 28 studies of which all presented data-driven findings. We defined the temporal workload of an IDS as the time delay between actual user intent and its detection, including the time required for the user to give the input and computational time. Most studies measured temporal workload by task duration (e.g., Zhang et al., 2019) or task speed by means of blocks per minute in the standardized Box and Block Test (e.g., Yurkewich et al., 2020b), therefore not only measuring the actual temporal workload of the IDS, but also including the inherent mechanical delay of the ULO and the time required for conducting the task. This is therefore only a valid option to rate temporal workload if these two parameters are approximately constant, i.e., when used to compare two different IDS used in combination with the same ULO and for the same task. Other studies have only measured the delay as the computational time between signal acquisition and classification or movement onset of the ULO (e.g., Delijorge et al., 2020), or the minimum possible time between two consecutive movements (e.g., Ortner et al., 2011). Some studies rated the temporal workload in term of the participant's subjective perception through non-standardized feedback (e.g., Ngeo et al., 2013), or through the NASA-TLX questionnaire by Hart and Staveland (1988) (e.g., Badesa et al., 2020).

#### 3.2.3. Ease-of-Use

The ease of use was assessed in 21 studies, of which nine were based on indirect findings. Since there is no formal definition of the ease-of-use, for this review, it was loosely defined as how easy the user found controlling the device using the IDS, i.e., it sums up whether they managed to use it with few explanations and low mental or physical workload. In literature, the term “intuitiveness” is sometimes used interchangeably with “self-explanatory” (Mohs et al., 2006), “familiar” or “using readily transferred, existing skills” (Raskin, 1994) and thus, in the broader sense, also relating to “easy to use.” However, a uniform definition of the term has not been established yet (Naumann et al., 2007). Therefore, for studies reporting “intuitiveness,” we interpreted from the given context whether the information was related to ease-of-use.

The indirect findings related to ease of use were mostly based on observations whether the users managed to use the IDS without problems (e.g., Ambrosini et al., 2014a) or without further instructions (e.g., Park et al., 2019). Data-driven findings were based on direct qualitative user feedback (e.g., Xing et al., 2008) or reported quantitatively with different scales and questionnaires including a custom numbered rating scale (e.g., Bermúdez i Badia et al., 2014), Usefulness, Satisfaction, Ease-of-use (USE) questionnaire by Lund (2001), the Quebec User Evaluation of Satisfaction with Assistive Technolgy (QUEST 2.0) questionnaire by Demers et al. (2002) (e.g., Yurkewich et al., 2020a) or the System Usability Scale (SUS) by Brooke (1996) (e.g., Shafti and Faisal, 2021).

#### 3.2.4. Learnability

A total of 16 studies assessed learnability, five reporting indirect statements. The learnability describes how much practice is required to be able to use the IDS appropriately or what influence prolonged use of an IDS has on the achieved performance. The included studies have described the learnability by three different metrics. Most of the studies did report how much practice participants needed until they were able to control the device according to their intent with acceptable performance. For indirect statements, this was done by observation (e.g., Hennig et al., 2020), data-driven findings were supported by measuring the required practice time (e.g., Yurkewich et al., 2020b) or by the participant's subjective perception on the ease of learning on a customized Likert-scale (e.g., Yap et al., 2017b) or as a subcategory on the SUS (e.g., Shafti and Faisal, 2021). Others reported the learning effect, e.g., the improvement of performance, which was observed for the same users after repeated use of the IDS (e.g., Webb et al., 2012). A small number of studies have also investigated learnability by comparing the performance of experienced and inexperienced users (e.g., King et al., 2014).

#### 3.2.5. Robustness

In contrast to the reliability, the robustness describes how an IDS performs under varying conditions, e.g., invalid user inputs, or stressful or changing environments. Some studies did assess the robustness analogously to the reliability by measuring success or error rates under varying conditions, e.g., with changing arm positions (Park et al., 2020), when distracting the participant (Ortner et al., 2011), or when the ULO is used with or without arm support (Park et al., 2019). Others provided a qualitative indication of the robustness of the IDS by identifying factors that do or do not influence its performance (e.g., Siu et al., 2018).

#### 3.2.6. Simplicity of Setup

Simplicity of IDS setup was discussed in seven studies. Six studies reported indirect, qualitative statements related to donning and doffing (Dwivedi et al., 2019), sensor placement (Meeker et al., 2017), or ease of calibration (Pedrocchi et al., 2013). One study assessed the donning and doffing process of the overall system systematically, but not of the IDS specifically (Lambelet et al., 2020).

#### 3.2.7. Enjoyability

Enjoyability was reported in five studies. The enjoyability sums up the user's mood, motivation, or frustration while using the IDS. One study reported enjoyability from indirect statements based on observation of the participants (Delijorge et al., 2020). The other studies reported enjoyability in terms of general qualitative direct user feedback (Ortner et al., 2011), numbered rating scales reporting “perceived fun” (Bermúdez i Badia et al., 2014), a visual-analog scale rating “mood” and “motivation” (Chowdhury et al., 2018) or frustration as subsection of the NASA-TLX (Badesa et al., 2020).

#### 3.2.8. Physical Workload

The physical workload was assessed in three studies. The reported physical workload provides an indication of how physically demanding the generation of a command is to the user. One study assessed physical demand using the NASA-TLX (Badesa et al., 2020) and one reported qualitative user feedback about the effort of use (Ambrosini et al., 2014a). The third study made an indirect statement about the appropriateness of the IDS in terms of the user's tendency to fatigue easily (Park et al., 2019).

#### 3.2.9. Mental Workload

Three studies assessed mental workload of the IDS. The mental workload describes how much mental/cognitive effort the user perceives or needs to exert while using the IDS. One of the included studies assessed mental demand using the NASA-TLX (Badesa et al., 2020), and two studies assessed the mental fatigue or exhaustion either by subjective comparison to other IDS (Soekadar et al., 2015) or on a visual-analog scale (Chowdhury et al., 2018).

#### 3.2.10. Naturalness

Two studies, both based on indirect statements, assessed naturalness. The naturalness describes how “natural” controlling the ULO by the specific IDS feels, compared to the normal, physiological initiation of the assisted or augmented movements. In contrast to what was described in the “ease-of-use”-section, the term “intuitive” is sometimes also used interchangeably with “natural” or “subconscious” (Lobo-Prat et al., 2014). Therefore, for studies that reported intuitiveness, we interpreted from the given context whether the information was related to naturalness. Both studies described their IDS qualitatively as “intuitive” (Kooren et al., 2016) or “more intuitive” compared to another IDS (Park et al., 2020).

#### 3.2.11. Comfort

Comfort were assessed in two studies, defined as the physical comfort or ergonomics perceived by the users during use. Both studies reported comfort based on qualitative user feedback, either in general (Delijorge et al., 2020), or related to specific aspects related to ergonomics, such as weight or obstruction of movement (Hennig et al., 2020).

#### 3.2.12. Cost

With only two assessments, cost (together with naturalness and comfort) was the least assessed attribute. Costs were defined as the financial effort to acquire or maintain usage of an IDS. However, both included studies discussed only acquisition, either as absolute price of the overall system (Webb et al., 2012) or relative to the performance of the IDS, i.e., cost-effectiveness (Araujo et al., 2021).

### 3.3. Types of Intention Detection Strategies

#### 3.3.1. Muscle Activation

Using electromyography (EMG), i.e., electric signals generated during muscle activation, was the most commonly used IDS, being used in 40.2% of the studies. Measuring the EMG signal as IDS has clear benefits for applications in daily life. The signal acquisition is relatively easy and feasible with standard commercially available hardware and the emerging use of dry, wireless electrodes allows their integration into wearable armbands such as the Myo armband (Thalmic Labs, Kitchener, Ontario, Canada; Meeker et al., 2017; Mohammadi et al., 2018; Park et al., 2019, 2020; Lambelet et al., 2020; Yurkewich et al., 2020a) or sleeves (Dwivedi et al., 2019), enabling very simple donning and doffing. However, EMG signals also have some inherent limitations. They are not robust against changing electrode placement, the electrical impedance of the skin, sweat, or muscular fatigue (Hameed et al., 2020). Further, for some users, EMG activation patterns might not be sufficiently strong or reproducible for effective intention decoding (Riley and Bilodeau, 2002; Park et al., 2020).

Purely binary or proportional controllers were used to control hand orthoses (DiCicco et al., 2004; Fujita et al., 2016; Dunaway et al., 2017; Lince et al., 2017; Yap et al., 2017a; Fardipour et al., 2018; Wang et al., 2018; Gerez et al., 2019, 2020; Yoo et al., 2019; Bos et al., 2020; Nam et al., 2020; Yurkewich et al., 2020a), and wrist (Yoo et al., 2019; Lambelet et al., 2020; Nam et al., 2020), elbow (Ambrosini et al., 2014a; Bermúdez i Badia et al., 2014; Fujita et al., 2016; Dunaway et al., 2017; Koh et al., 2017; Nam et al., 2020), or shoulder orthoses (Ambrosini et al., 2014a; Fujita et al., 2016; Scheuner et al., 2016; Zhou et al., 2021). Three studies did not provide unambiguous information about the used control method (Pedrocchi et al., 2013; Mohammadi et al., 2018; Rose and O'Malley, 2019). A total of 15 studies used pattern recognition-EMG techniques to control hand orthoses (Ngeo et al., 2013; Kawase et al., 2017; Meeker et al., 2017; Siu et al., 2018; Burns et al., 2019; Dwivedi et al., 2019; Farinha et al., 2019; Lu et al., 2019; Park et al., 2019, 2020; Secciani et al., 2019; Zhang et al., 2019), and wrist, elbow, or shoulder orthoses (Kiguchi, 2007; Kawase et al., 2017; Kilic, 2017; Lotti et al., 2020). To do so, up to 12 EMG channels were used (Kiguchi, 2007), controlling up to a maximum of six different states (Dwivedi et al., 2019; Lu et al., 2019; Zhang et al., 2019). Various studies reported conventional, i.e., binary or proportional, EMG control as being easy to understand or use (Gerez et al., 2019; Yoo et al., 2019; Yurkewich et al., 2020a). Yurkewich et al. (2020a) further reported an average reliability of 84.7% (*n* = 9, stroke) to control three states of a 1-DOF hand orthosis using the Myo armband with eight electrodes. Bos et al. (2020) found a strong training effect in a force tracking task using a proportionally controlled 1-DOF hand orthosis (*n* = 1, duchenne muscular dystrophy). For pattern recognition-EMG techniques, primarily, reliability, robustness, and temporal workload were reported. Siu et al. (2018) determined how much faster the ULO can be controlled by EMG compared to exerted forces. They found average anticipation times, i.e., the time that EMG detects intent earlier than the increase of pressure measured at the thumb, between 190 and 290 ms. Lotti et al. (2020) achieved a delay below 53.8 ms in 95% of trials, also suggesting that this IDS might be faster than other approaches based on interaction forces. The reported reliability varied largely between and within studies from 40% (six states, *n* = 1, stroke; Lu et al., 2019) up to 96.4% (six states, *n* = 6, neurologically intact; Zhang et al., 2019). The achieved reliability has been shown to depend on a number of factors, e.g., chosen classifier (Dwivedi et al., 2019), impairment of the subject (Lu et al., 2019), or co-activation of surrounding muscles (Park et al., 2019). Although some studies have shown very high reliability, this large variability and dependence on many factors indicate that pattern recognition techniques might not yet be robust enough for applications in daily living. For the other studies, no relevant usability attributes related to this IDS were reported.

#### 3.3.2. Muscle Contraction

Various approaches have been explored to measure mechanical muscle contraction: either by means of low-frequency vibrations of the muscle fibers, i.e., mechanomyography (MMG) (Courteville et al., 1998; Ibitoye et al., 2014), or by measuring a change in muscular stiffness patterns, i.e., force myography (FMG), also referred to as kinetic imaging, muscle pressure mapping, pressure distribution mapping, or tactile myography (Xiao and Menon, 2019). The operating principle and target application of FMG is very similar to EMG. Therefore, these IDS share some benefits, i.e., physiological operating principle and the possibility to integrate the sensors into wearables (Kudo et al., 2014; Dwivedi et al., 2019), but also related inherent limitations, i.e., sensor placement or muscular fatigue. Sensors mechanically measuring the muscle contraction are robust to moisture and not susceptible to electromagnetic noise (Fajardo et al., 2019; Xiao and Menon, 2019).

No studies were found using MMG, but six studies used FMG as IDS for ULO. Dwivedi et al. (2019) integrated five resusable FMG sensors and three EMG sensors into a textile sleeve to differentiate between six grasp types of a soft robotic glove. Yap et al. (2016) integrated three FMG sensors into a textile band worn on the contralateral forearm to differentiate between finger flexion and extension. Moromugi et al. (2013) and Kim et al. (2012) used pressure sensors on the fore- or the upper arm to control one DOF of a hand or elbow orthosis, respectively. Fajardo et al. (2019) presented a system using two optical fiber sensors which measure the muscle deformation by the displacement of the fibers. A special application of FMG as IDS was presented by Kudo et al. (2014), where they used FMG signals from the temporalis muscle. They integrated soft force sensors into a headphone-like interface to trigger the grasp of a 1-DOF hand orthosis.

Dwivedi et al. (2019) achieved classification accuracies above 87% for six grasp types, requiring < 0.12*s* processing time (*n* = 2, impairment not reported). Yap et al. (2016) compared the temporal workload in terms of task time between using a button and FMG, where they found a 2% higher task time for FMG (*n* = 1, neurologically intact). For the remaining studies, no data about the usability of their IDS was reported.

#### 3.3.3. Upper Limb Movement

Intention can be interfered from joint rotation or kinematics of upper limb segments, allowing natural and easy operation. However, this IDS depends on sufficient residual upper-limb function, thus being primarily applicable for devices targeting augmentation of neurologically-intact users, or orthoses to support people with limited, but residual upper limb functionality. For people with more severe impairments or full paralyses, these IDS are not a feasible alternative.

Seven studies used bending sensors to detect finger (Ab Patar et al., 2014; Popov et al., 2017; Xiloyannis et al., 2018; Park et al., 2019) or wrist motion (Kaneishi et al., 2019; Rose and O'Malley, 2019) to control hand orthoses, or wrist motion to control a shoulder-elbow-orthosis (Koo et al., 2009). Four studies detected residual upper limb movement by using inertial measurement units (IMUs) attached to a segment of the upper limb. Song et al. (2012) and Wang et al. (2020) attached IMUs to the forearm and the upper arm to control elbow and shoulder orthoses, respectively. In both cases, the initiated movement measured by the IMU was directly converted to the actuated movement of the ULO. Yurkewich et al. (2020b) attached a single IMU to the dorsal side of the hand and triggered opening and closing of a 1-DOF hand orthosis when the rotational velocity of the hand exceeded a predefined threshold. Zhou et al. (2021) used kinematics measured by IMUs at the upper arm and the trunk to control a 1-DOF industrial shoulder exoskeleton. Park et al. (2020) measured shoulder movement (i.e., a shrug) using a shoulder harness with integrated load cell. Although perceived as less natural than EMG, they presented this IDS as an alternative for users who can't modulate sufficient EMG signals.

Ab Patar et al. (2014) and Koo et al. (2009) both qualitatively reported ease of use of the IDS. Yurkewich et al. (2020b) compared their IMU-based strategy to a conventional button, where they found lower temporal workload, but also longer practice needed and lower reliability than for the button (*n* = 11, stroke). Five participants reported that they would prefer the IMU- over the button mode, if the former would work more reliably. Zhou et al. (2021) compared their IMU-based strategy to control by threshold-based EMG. They found the former to be more reliable and robust, but slightly slower than the EMG based method (*n* = 8, neurologically intact). The remaining studies did not present any relevant usability attributes related to this IDS.

#### 3.3.4. Isometric Force Generation

Similar to joint rotation or UL kinematics, the intent can be detected by measuring isometric forces when initiating a movement of the upper limbs. Since the initiation of the desired motion is (partly) restricted by the mechanical structure of the ULO, isometric forces or torques will be measurable between upper limb and ULO or between ULO and the physical environment. However, same as for IDS based on upper limb movements, this IDS depends on sufficient residual upper-limb function.

In the case of hand orthoses, force, or torque sensors were attached to the tips of the actuated fingers to control grasping proportionally or by a threshold-based trigger (Xing et al., 2008; Song and Chai, 2013; Heo and Kim, 2014; Ma et al., 2016; Prange-Lasonder et al., 2017; Chowdhury et al., 2018; Siu et al., 2018; Triolo et al., 2018; Xiloyannis et al., 2018; Park et al., 2019; Zhou et al., 2019; Hennig et al., 2020; Sandison et al., 2020). Hong et al. (2019) have triggered hand orthosis opening and closing by a strain gauge attached to the non-actuated ipsilateral thumb. For wrist, elbow or shoulder orthoses, the sensors were placed between the respective upper arm segment and mechanical structure of the ULO (Sasaki et al., 2005; Kiguchi, 2007; Lee et al., 2008; Yonezawa et al., 2013; Kooren et al., 2016; Kapsalyamov et al., 2019).

Using a strategy where the hand orthosis closed when the pressure between finger tips and objects to be grasped exceeded a predefined threshold, Zhou et al. (2019) and Hennig et al. (2020) found false positive rates of 9.9% (*n* = 3, spinal cord injury) and 6.9% (*n* = 3, neurologically intact), respectively. Other studies reported qualitatively that the IDS was intuitively controlled (*n* = 1, neurologically intact; Kooren et al., 2016), easy to use (*n* = 3, neurologically intact; Xing et al., 2008), and that the sensors directly embedded in the ULO allow for quick and easy setup (Sandison et al., 2020). Park et al. (2019) compared multimodal control (pattern recognition-EMG, pressure sensor on thumb, bending sensors on each finger) to pure EMG control of a 1-DOF hand orthosis and found higher reliability but higher temporal workload for the multimodal control when used without passive arm support (*n* = 4, stroke). For the other studies, no relevant usability attributes related to this IDS were reported.

#### 3.3.5. Brain Activity

Brain-computer interface (BCI) research has been a focus of interest for the past decades, maturing the technology from simple communication devices in controlled laboratory environments to more practical application for rehabilitation (Mane et al., 2020) or assistive technologies in daily life (Millán et al., 2010; Kübler, 2020). For example, at the CYBATHLON 2016, pilots used four commands (i.e., three active commands and one “rest” command) to maneuver a BCI-controlled avatar through a virtual racetrack (Riener, 2016; Novak et al., 2018). Undeniably, the most significant advantage of BCIs is that they do not rely on any residual motor control. In the most severe case of paralysis, for people with total locked-in syndrome, a neurological disorder that results in a complete inability for any muscular movement, BCIs represent the sole viable approach for communication and interaction with their surrounding (Kübler, 2020). Still, it has been shown that BCIs fail to correctly detect the desired state for an estimate of 20% of people, presumably due to the complete inability of the users to modulate respective brain signals, so-called BCI illiteracy (Allison et al., 2010; Blankertz et al., 2010). This apparently inevitable limitation effectively excludes a large subset of potential users of BCI. In terms of daily life applicability, the rather complicated setup and hardware required might not yet be sufficiently easy to use for users without extensive technical knowledge (e.g., calibration or donning and doffing).

The most common non-invasive technique to measure brain activity is electroencephalography (EEG), i.e., using electrodes placed on the scalp to measure the electrical activity of groups of nerve cells from the cerebral cortex. For multichannel measurements, arrays of electrodes, e.g., incorporated in wearable caps, are used to ease donning and doffing and ensure the precision of placement (Teplan, 2002). On the highest level, BCIs can be grouped into endogenous and exogenous techniques. In endogenous techniques, the subject can actively operate the BCI at free will by performing (motor execution) or imagining to perform (motor imagery) a specific task. A meta-analysis by Hétu et al. (2013) has shown that motor imagery activates fronto-parietal, subcortical and cerebellar regions of the brain. However, although there are some regions which are involved in motor execution as well as motor imagery, the latter does not consistently activate the primary motor cortex (Hétu et al., 2013). Therefore, a clear distinction between these two strategies is required. In contrast, in exogenous techniques, the brain activity elicited by external stimuli is used to operate the BCI (Nicolas-Alonso and Gomez-Gil, 2012). These technique use event-related potentials (ERP) based on changes in the EEG signal evoked in response to external sensory, motor, or cognitive events (Sur and Sinha, 2009), e.g., focusing on flickering icons on a screen (Pedrocchi et al., 2013), or steady-state visually evoked potentials (SSVEP), based on EEG response evoked by visual stimuli at specific frequencies (Guger et al., 2012), e.g. focusing on two light-emitting diodes flickering at different frequencies (Ortner et al., 2011).

From all included studies, 16 used EEG-based systems based on motor imagery (Pfurtscheller et al., 2000; Webb et al., 2012; Xiao et al., 2014; Soekadar et al., 2015; Bi et al., 2017; Cantillo-Negrete et al., 2018; Kapsalyamov et al., 2019; Zhang et al., 2019; Badesa et al., 2020; Araujo et al., 2021), motor execution (Fok et al., 2011; King et al., 2014; Lee et al., 2017; Chowdhury et al., 2018), SSVEP (Ortner et al., 2011), or ERP (Pedrocchi et al., 2013; Delijorge et al., 2020) to control the ULO. One included study by Lee et al. (2017) exploited brain activation not directly by EEG, but indirectly by measuring hemodynamic responses, using functional near-infrared spectroscopy (fNIRS), i.e., the varying concentration of oxygen in the blood in activated nerve cells in the cerebral cortex (Naseer and Hong, 2015). They used an fNIRS-setup in a motor-execution study to trigger the opening and closing of a 1-DOF hand orthosis. All BCI studies differentiated only between two states, e.g., “open/close” or “confirm/reject.”

From the 17 studies, 15 assessed reliability, achieving classification accuracies in a controlled laboratory environment between 70% (*n* = 8, neurologically intact; Cantillo-Negrete et al., 2018) and 91.5% (*n* = 1, neurologically intact; Araujo et al., 2021). However, the variance in performance could not be traced back to an individual factor, but may be influenced by many. The included studies varied largely in terms of, e.g., the number and type of electrodes used [from one (Ortner et al., 2011) up to 40 (Zhang et al., 2019)], the type of signal modulation (motor imagery, , motor execution , SSVEP , or ERP ), unrejected motion artifacts, or the chosen classification approach. However, BCI performance also largely depends on the user's ability to modulate brain signals of sufficient quality (Allison et al., 2010). Practice and experience of the user are widely considered as influential aspects to promote BCI performance (Millán et al., 2010), as underlined by Webb et al. (2012), and King et al. (2014) in their motor imagery-based study who both showed higher accuracies in the second session (*n* = 4, neurologically intact) or for BCI-experienced compared to BCI-naive users (*n* = 6, neurologically intact), respectively. On the contrary, Ortner et al. (2011) did not find differences in reliability between experienced and naive participants and between sessions in an exogenous application (*n* = 6, neurologically intact). Ten studies further assessed the temporal workload of EEG-based BCIs in the order of 2–10 s (Ortner et al., 2011; Webb et al., 2012; King et al., 2014; Xiao et al., 2014; Bi et al., 2017; Zhang et al., 2019). The fNIRS-motor execution study by Lee et al. (2017) reported 78% classification accuracy and measured time from signal acquisition to movement onset as 5.84 ms (*n* = 6; neurologically intact).

#### 3.3.6. Tongue Movement

Movement of the tongue has been used to control computers or assistive devices for people with severe motor disabilities (Struijk, 2006). In general, since there is no evident natural relation between tongue- and upper-limb movement, users need to learn which inputs result in the desired actions, requiring high initial mental effort. Many conditions leading to upper-limb impairment do not affect tongue movement, making the approach feasible for a broad group of target users with impaired upper-limb function. However, the need for a distinctive tongue movement makes using the device and talking simultaneously impossible, potentially restricting the use for some applications in daily life.

Tongue movement was used in only a single study. Kim et al. (2013) used a headset that positions four magnetic sensors near the user's cheek to trace the movement of a small magnetic tracer temporarily glued to the tip of the user's tongue. The position of the tracer in the oral cavity was then mapped to the angle of an actuated 1-DOF wrist orthosis for rehabilitation. The study compared three control methods (tongue movement binary up/neutral/down, proportional left/right, or proportional anterior/posterior) in a trajectory tracking task, where participants achieved highest tracking accuracies with the proportional left/right control (*n* = 3, neurologically intact).

#### 3.3.7. Eye Movement

Eye movement plays a crucial role in human motion planning by gathering information about the object or the environment to be manipulated before initiating the movement (Land, 2006). Visual input, e.g., from tracking the user's eye motion or gaze point, can thus be used to guide the movement of the upper limb supported by the ULO for reaching or grasping tasks (Cognolato et al., 2018). Since most neurological deficits resulting in limited upper limb functionality do not affect eye movement, eye-tracking is a feasible IDS for a broad target population. Different eye-tracking techniques exist, two prominent examples being video-oculography, measuring the position of the eye by the corneal reflection with video cameras or electrooculography (EOG), i.e., measuring the difference in the electrical potential between the retina and cornea through electrodes placed in the area around the eye (Barea et al., 2002). The natural relationship between eye movement and movement intention and comparably simple calibration methods (Pedrocchi et al., 2013) makes eye movement-based IDS easy to learn and to use to control ULO. However, an inherent challenge of this IDS is to differentiate between non-specific visual scanning and actual movement intent. Thus, eye-tracking is often used in combination with other IDS.

Three of the included studies used IDS based on eye movement. Soekadar et al. (2015) proposed an EOG-EEG-based system, where the users could look to the left or to the right to approve or reject the EEG-based movement decisions. Zhang et al. (2019) used EOG to detect eye movements to the left or right and double blinks to select between two grasp types or switch between different multimodal IDS (EOG, EMG, or EEG). The third included study, Shafti and Faisal (2021) used an IDS involving corneal reflection measurements using eye-tracking glasses and object recognition. They triggered the movement of the ULO when the user fixated a specific area of the object to be grasped.

When using EOG in combination with EEG, Soekadar et al. (2015) found a significant improvement in reliability and participants reported lower mental workload and higher ease-of-use, compared to only using EEG (*n* = 5, neurologically intact, *n* = 1 brachial plexus injury). Zhang et al. (2019) found that, after a training phase of under 2 min, participants were able to use the EOG-based IDS with an accuracy of 94.2% and an average temporal workload of 1.2 s per action (*n* = 6, neurologically intact). For the object recognition technique used by Shafti and Faisal (2021), they achieved a 96.6% success rate at first attempt and all participants rated learnability and ease-of-use between 3 and 5 out of five points on the SUS (*n* = 5, neurologically intact).

#### 3.3.8. Speech

Nowadays, using voice control is predominantly known for controlling consumer electronics such as smartphones or home automation systems. However, it has also been used in medical technologies such as wheelchairs (Simpson and Levine, 2002), surgical robots (Zinchenko et al., 2017), or ULO. Similar to eye movement, voice control is feasible for a broad target population with a wide range of impairments, as long as speech is not drastically affected. The number of distinguishable states is theoretically infinite, practically limited only by the computational power, the used software, and the potential need for internet connectivity for recognition. However, in a noisy environment with interfering sources of sound or voices, the performance accuracy may be affected. For other specific scenarios, e.g., in an otherwise quiet environment such as the theater or during spoken conversations with other people, some users may find the need to pronounce distinct words disturbing.

Voice control was used in four studies to trigger the movement of the ULO, where the user needed to pronounce a specific word to actuate three (Ochoa et al., 2009; Kim et al., 2018), six (Dalla Gasperina et al., 2019), respectively seven (Wang et al., 2018) states of the ULO. Each word was assigned to a specific action of the ULO, e.g., corresponding to opening and closing of a hand orthosis (Ochoa et al., 2009) or controlling the position end-effector of a shoulder-elbow-wrist exoskeleton by words corresponding to the six main directions in the cartesian space (Dalla Gasperina et al., 2019). Wang et al. (2018) is the only study assessing usability aspects of voice control. They found correct recognition rates above 94% with recognition times between 47 and 50 ms (*n* = 2, impairment not reported).

#### 3.3.9. Manual Triggers

Manual triggers, i.e., buttons, joysticks, or touchscreens, are often the first IDS of choice for ULO. Most users are accustomed to the use and look of these interfaces since these are commonly known from other conventional devices in daily life. Although the relationship between the operation principle of these IDS and their initiated action is not natural, they are mostly self-explanatory and require minimal to no training or calibration. They are generally easy to use and provide high reliability and robustness since they are not dependent on any physiological signal or complex processing. The number of states or actions of the ULO can be increased arbitrarily, e.g., by increasing the number of buttons, however only at the expense of increased cognitive workload for the user. Manual triggers are commercially available in many variants, and can be placed anywhere, e.g., directly on the ULO, on a body part of the user, or on the table, enabling easy adaptability to the users' capabilities. However, using touchscreens, joysticks, and—depending on their size and placement—buttons usually requires some residual function and movement accuracy in the upper limbs from the users. Further, using an upper limb segment—in most cases the fingers or the hands—to control the trigger restricts their use for bimanual or simultaneous tasks, which considerably limits the applicability in daily life tasks.

In 21 studies, conventional manual triggers such as buttons/switches (Ochoa et al., 2009; Pedrocchi et al., 2013; Ambrosini et al., 2014a; Yap et al., 2016, 2017a; Meeker et al., 2017; Fardipour et al., 2018; Otten et al., 2018; Butzer et al., 2019, 2021; Farinha et al., 2019; Correia et al., 2020; Gerez et al., 2020; Muehlbauer et al., 2021), joysticks (Hasegawa and Oura, 2011; Dalla Gasperina et al., 2019; Ismail et al., 2019; Tiseni et al., 2019), or touchscreens (Yap et al., 2017b; Mohammadi et al., 2018; Sandison et al., 2020) have been used.

Presumably due to the simplicity of these manual triggers and the previous familiarity of most users with them, none of these studies assessed usability attributes specifically only related to these IDS. Instead, many of the included studies used these inputs as baseline to compare to alternative IDS, such as EMG or IMU-based systems (Ambrosini et al., 2014a; Yap et al., 2016; Meeker et al., 2017; Yurkewich et al., 2020b). Other studies used them in combination with other IDS to control only a subset of actions, e.g., to select grasp type before using a different IDS as trigger (Gerez et al., 2020).

## 4. Discussion

This scoping review provides a comprehensive overview of studies evaluating non-invasive IDS in combination with ULO for applications in daily life. Further, it describes methods of usability evaluation used in these studies. By including only studies that involved human participants in the evaluation, a focus was set on the appropriateness of the IDS from a user point of view. The basic operation principles, as well as the usability of the proposed IDS, were reviewed and discussed. In addition, evidence of their appropriateness for different target users, type of devices, and usage scenarios were gathered, considering predominant usability attributes. This work extends existing reviews in the field (Lobo-Prat et al., 2014; Chu and Patterson, 2018; du Plessis et al., 2021) by refreshing the current state of the art in IDS (as reflected by more than half of the included studies being published within the last 3 years), as well as by analysing these under a different angle, giving less priority to the technical aspects, but focusing on the usability evaluation of IDS with real users.

### 4.1. Considerations When Selecting an Appropriate IDS

#### 4.1.1. IDS Presented in This Scoping Review

The included studies revealed the breath of IDS that were used to control ULOs in the literature. On the level of the physiological phenomenon (level 2), IDS were presented based on muscle activation, muscle contraction, force exertion, residual upper limb movement, brain activity, tongue and eye movement, speech, and manual triggers. A previous review conducted by Lobo-Prat et al. (2014) investigated IDS for all active movement assistive devices such as prostheses and orthoses for the upper and lower limbs or powered wheelchairs. They presented some IDS which were not found in the included studies of this review: based on brain activity [magnetoencephalography (MEG), functional magnet resonance imaging (fMRI)], muscle contraction [MMG, sonomyography (SMG)], and head movement. Some of these IDS are not suitable for ULO since they are either not wearable (fMRI, MEG) or too cumbersome to use (SMG) (Lobo-Prat et al., 2014). MMG however, although it has been used only rarely and primarily in upper limb prosthetic control so far (Silva et al., 2005; Woodward et al., 2015), might be a viable option for ULO as well. It was already used by Antonelli et al. (2009) to control lower limb orthoses, allows wearability, and has similarities in signal acquisition and processing to EMG and FMG. IDS based on head movement are primarily used for powered wheelchair control (Kupetz et al., 2010; Solea et al., 2019). However, this IDS is primarily used for people for whom head movement is one of the sole possible movements. Thus, although generally possible, this might not be the most desirable option to control ULOs.

Further, there are other studies presenting IDS which were neither presented in Lobo-Prat et al. (2014) nor in this review. Kojima et al. (2017) and Cunningham et al. (2018) presented a supernumerary robotic arm and thumb, respectively, which were controlled through toe or ankle motion. To control flexion and extension of a wearable supernumerary finger, Hussain et al. (2017) embedded EMG sensors in a baseball cap to detect EMG signals from the frontalis muscles, which are contracted by moving the eyebrows upwards. Similarly, Kocejko (2017) used binary one-channel EMG to detect contractions of the temporal is muscle while tightening the jaw to select from three gestures of an arm prosthesis. Commercially available sip-and-puff systems were used for wheelchair control (Grewal et al., 2018) or communication devices (Jones et al., 2010) only. Although all these IDS are relatively rarely used due to their limited applicability to broader contexts of use, these could be additional options for specific ULO beyond the ones presented in this work.

#### 4.1.2. Avoiding the Restriction of Other Body Functions

This review has identified the strengths and weaknesses of IDS used in combination with ULO in specific contexts of use. As depicted by the vast majority of studies using IDS sourcing from the targeted upper-limb segments, the use of signals related to the physiological movement execution, whenever possible, is preferred by most users and researchers. The rationale behind linking the actual intention to its physiological motor consequence is two-fold: the IDS does not restrict other body functions, and neuroplasticity, i.e., the reorganization of the central nervous system in response to intrinsic or extrinsic stimuli (Cramer et al., 2011), may be enhanced. Therefore, for contexts of use where recovery is considered realistic and the primary target, using physiological signals as IDS may promote a rehabilitative effect. Based on this, we expected that other IDS based on non-physiological movement, e.g., manual triggers or IDS sourcing from other body parts than the targeted upper-limb segments or the brain, were primarily considered when the physiological signals are either not sufficiently strong, or if the ULO targets assistive rather than therapeutic applications. The found distribution of the intended scenario showed tendencies which strengthen this assumption, however they were not sufficiently strong to make an unambiguous conclusion.

By far, the IDS exploited the most is EMG. However, EMG showed some challenges and inherent limitations, currently restricting its transfer to real-life applications. These limitations encouraged researchers to explore alternative IDS, such as mechanically measuring muscle contraction or residual movement. However, evidence demonstrating an absolute superiority of these IDS over EMG is still scarce. For users who do not have sufficient residual muscle activity and motor function in their upper limbs, IDS sourcing from other body parts are an alternative. The wide variety in terms of the source of the signal offers adaptability to the user's individual capabilities and preferences. However, except for inputs from the brain, these IDS do not resemble the physiological movement generation. Thus, IDS sourcing from other body parts than the ULO are not perceived as natural and require the users to learn which input—usually a specific motion or activation of a muscle—results in the desired movement. Further, their biggest drawback is that they can only be used at the expense of restricting other body functions during use. Therefore, for these IDS, it is crucial to individually weigh the impact such a restriction might have against the potential benefit the ULO would provide in the intended usage scenario. Unquestionably, in theory, BCIs offer a vast potential as the most natural and most broadly applicable IDS. However, the current state of non-invasive BCI research for ULO has not yet managed to overcome the usability hurdle (i.e., in terms of robustness, temporal workload, and simplicity of setup) to be used in real applications outside the controlled laboratory environment.

#### 4.1.3. Balancing Performance and Complexity

In many studies, the rationale behind the usability attributes assessed is not always explicitly or sufficiently stated. While some requirements are unambiguously given by the application (i.e., number of states to be controlled or functional capabilities by the targeted user), others might call for a more in-depth focus on the user and the intended usage scenario. Unfortunately, in most studies, information on the latter is provided on a very high level or lacks completely. A central decision when selecting an appropriate IDS is the trade-off between high performance (i.e., high reliability and robustness) and low complexity (i.e., high ease of use and learnability and low workload for the user). As a guiding principle, the IDS achieving the highest usability are those which are as simple as possible but as complex as needed to achieve the required performance. In commercial devices, mostly incorporating a relatively low number of states, presumably simpler IDS (e.g., push-buttons or conventional EMG control methods) are currently used. The same reliance on simpler solutions can also be observed for assistive technologies provided to persons with sensorimotor disabilities for use in daily life. However, in-depth evaluations of the usability of simple IDS might not have been considered worth investigating by scientific researchers. This potential bias toward more complex IDS leads to the speculation that simpler IDS are underrepresented in the field (and in this review) in comparison to their apparent high usability for commercial devices. However, especially for applications requiring multiple states to be controlled simultaneously, the trade-off between complexity and performance is often harder to find. The required performance seems to be very challenging to achieve with the choice of a single IDS, which is usually simple but only applicable to a low number of states or very complex itself, i.e., pattern recognition techniques in EMG or BCI research. Thus, many studies combined multiple different IDS for a single application. Such multimodal approaches have been successfully implemented to improve the overall functionality of the IDS. Some studies used multiple IDS to increase the reliability of controlling a single state (e.g., opening/closing of a 1-DOF hand orthosis using EEG and EOG; Soekadar et al., 2015), while others used multiple IDS to simultaneously control one state each (e.g., selection of movement type by buttons and movement trigger by EMG signals; Gerez et al., 2020). However, although each implemented IDS separately might be comparably simple to use, simultaneously providing inputs sourcing from different body parts may become cognitively challenging. Although not included in the scope of this review, promising approaches to tackle this issue are shared-control methods (Losey et al., 2018). These methods combine high-level control by the user with autonomous low-level control by the robots, e.g., through camera-based object recognition (Markovic et al., 2015; Fajardo et al., 2018) to relieve the physical and mental burden from the users, while still leaving them in control.

### 4.2. Considerations When Evaluating an IDS

#### 4.2.1. Tendency to Prefer Objective Over Subjective Attributes

By determining the frequency of assessment of the predefined usability attributes, we found that the majority of evaluations focused on attributes which could be evaluated objectively and quantitatively, e.g., reliability or temporal workload. Although specifically only including studies with human participants in this review, subjective or qualitative aspects, often acquired from user feedback or observation, were reported less frequently or not in a data-driven format. Overall, attributes assigned to the groups “effectiveness” or “efficiency” tended to be assessed more frequently than those assigned to the group “satisfaction.” Although this finding has to be treated with caution due to the unbalance in the total number of attributes per group, this might lead to the assumption that most evaluations highlighted rather technical than strongly user-focused aspects of the IDS. This coincides well with the initial observation of the technical focus of many existing literature reviews and our recent survey on wearable robotics usability evaluation (Meyer et al., 2021). While the technical performance is unarguably an essential prerequisite for meaningful use of an IDS, the impact of usability attributes with a stronger focus on the user such as satisfaction or perceived physical or mental workload should not be neglected. As an example, a systematic review investigating user needs for assistive technologies for the upper limbs after stroke by Ommeren et al. (2018) listed—among other attributes—comfort, donning/doffing, and setup (i.e., “simplicity” according to this review's definition) as relevant themes to achieve higher levels of user satisfaction and device acceptance. Yet, comfort and simplicity were only assessed in 3 and 7%, respectively, of the studies included in this work. The lower frequency of reporting qualitative attributes than quantitative ones could result from a publishing bias. Quantitative outcomes are often objectively verifiable, comparable and allow statistical analysis, allowing “high quality” evidence. However, in subjective user evaluations, only few attributes are *per se* quantitatively measurable. Therefore, many studies quantify the qualitative findings by assigning to them a ranked numeric value, allowing statistical analysis and comparability to other studies. However, the problem of subjectivity remains and thus potentially lowers the research interest in these findings. In summary, all these observations point to an important realization: the frequency of reporting of a usability attribute might not necessarily correlate with its importance for the users and the application of the IDS in daily life.

#### 4.2.2. Determining Appropriateness of an IDS

A recurring challenge when selecting an appropriate IDS is the fact that most studies compare their results to other studies to demonstrate superiority, but lack a clear benchmark to specify whether their results are actually good (and thus the IDS appropriate) or not. An illustrating example is the classification accuracy in EMG. While unarguably an accuracy of e.g., 90% is better than 86%, if achieved under comparable conditions, it is not clear whether 90% is sufficient. Thus, to properly be able to rate the acceptability of the reported accuracy values and of the IDS, an appropriate benchmark for acceptance should be defined. For brain-computer communication such as cursor control, Kübler et al. (2006) assumed a minimally required threshold of 70% accuracy. However, in ULO, erroneous actions can lead to serious safety issues, assumingly increasing the required threshold. This question was also discussed in the “hot coffee problem” (Ajiboye and Weir, 2005), a thought experiment describing a system with 99% accuracy. In a task-oriented manner, this would mean that users would spill hot coffee over themselves in 1 out of 100 trials—a safety risk that seems unacceptably high. Following that logic, an acceptably reliable and thus safe IDS would need to achieve performance accuracies similar to the non-impaired limb (Ajiboye and Weir, 2005), a value that has not yet been experimentally determined. Unfortunately, such benchmarks for usability attributes are impossible to define in a generalized manner since they largely depend on the context of use of the IDS. Instead, they would need to be customized to one specific context of use, e.g., by consulting target users. In addition to the common approach to compare the results to other studies or IDS, such benchmarks would allow for a standalone and objective rating of the appropriateness of an IDS for the targeted context of use.

### 4.3. Limitations of This Work

One limitation of this work is also one of the biggest hurdles in the field of usability evaluation of wearable robots in general: there's a lack of a common understanding or standardized definitions of usability attributes. Thus, the selection of attributes as well as their definitions were done based on the subjective experience and interpretations of the authors of this work. For some cases, these definitions might not match the one from the authors of the included studies, potentially leading to different subjective interpretations of the collected data. It should also be noted that studies which evaluated the IDS without human participants or without the ULO being actually worn by the participants were excluded. Although this was intended in the review protocol to focus on a more realistic real-life scenario, this might have excluded many potentially interesting IDS. Specifically, many IDS currently under development might not yet be at a sufficient level of technology maturity to be safe and robust enough for testing with human participants, despite having the potential to become highly usable and broadly applicable. The technology maturity of the IDS does presumably also have an impact on the assessed usability in the included studies. Thus, some of these IDS might have the potential to achieve higher usability in a future development stage. However, since the maturity of the presented technologies was often not reported or not reported systematically, this aspect could not be taken into account for the synthesis. Further, many studies assessed the overall device consisting of ULO and IDS as a single unit instead of two interacting but separate systems. Despite efforts to only include specific usability attributes only concerning IDS and exclusion of findings which were clearly influenced by the ULO, the possibility that some of the reported findings may be biased by the usability of the ULO itself, can't be ruled out. Lastly, the partially qualitative nature and the heterogeneity of the collected data did not allow an objective and systematic rating of the individual IDS, nor a statistical or meta-analysis. A more quantitative comparison between IDS would be more beneficial for an objective selection of IDS. However, the available data did not allow such an analysis.

### 4.4. Implications and Recommendations for Future Research

This review provides a comprehensive understanding of the evaluation practices and results for IDS used in combination with ULO. The collected data on specific usability attributes for a wide range of IDS and their respective applications to various contexts of use can serve as a catalog of solutions for technology developers needing to select an appropriate IDS for their application. Although this work's scope is focused on ULO, its conclusions may also apply for other devices sharing similar challenges, such as neuroprosthetics (Taylor et al., 2002; Ambrosini et al., 2014b; Fonseca et al., 2019), supernumerary limbs (Hussain et al., 2017; Cunningham et al., 2018), or prosthetics (Micera et al., 2010; Parajuli et al., 2019). The importance of a user- and application-specific selection of IDS for such devices is underlined by the finding that no IDS can be rated as being generally superior to another without specifying the detailed context of use, i.e., the type of device for which the IDS is intended, the target user's capabilities and preferences, as well as the targeted usage environment and the tasks for which the device should be used.

Based on the insights from this review, we propose four recommendations to technology developers in the field, related to the selection of IDS, as well as to the design of corresponding evaluation protocols:

1. When designing or selecting an IDS, researchers should carefully consider the detailed context of use in which the ULO is intended to be used. Accordingly, these considerations should be described in the respective publications to allow for an informed evaluation of the IDS with respect to the intended context of use.
2. To reach a broader target population, ULO should offer their users a selection of different IDS to choose from or to combine to accommodate for the user's individual capabilities, preferences, and usage scenarios. For example, while specific users might prefer voice control at home they might want to switch to a button to be able to control their ULO more quietly in a restaurant or at the cinema.
3. Based on the user requirements, appropriate protocols to evaluate the usability of an IDS should be set up. These protocols should combine different scales and methodologies and cover not only technical aspects related to the efficiency or effectiveness of the IDS but also critically take user satisfaction, e.g., obtrusiveness, simplicity for donning/doffing, or comfort, into consideration. Such a comprehensive user evaluation protocol would allow for better interpretation of the usability evaluation results, help to set benchmarks and set the findings from the evaluation in relation to potential implications for the overall device acceptance by the target users.
4. When evaluating specific usability attributes, IDS, and ULO should, whenever possible, be assessed as two separate entities, interacting with each other and with the user, instead of as a single unit. Such an independent evaluation would allow discovering the source of potentially arising design- or usability issues related to the IDS or the ULO more easily and earlier.

## 5. Conclusion

Choosing an optimal IDS for a given application remains a recurring challenge since it is highly dependent on many factors, such as the intended usage scenario and target user's capabilities, limitations and preferences. By providing a comprehensive overview and recommendations for future development, this work encourages technology developers in the field to administer a user- and application-specific selection of appropriate IDS for ULO. Such a selection would positively affect the usability of the overall device and thus the device acceptance by the target users, ultimately promoting the leap of such technologies out of research laboratories into the target user's homes to positively impact the quality of life of end-users.

## Author Contributions

JG, OL, and RG designed the aim and scope of the review. JG conducted the database search, performed the data extraction and the quality assessment, analyzed the review findings, and wrote the manuscript. JG, JD, and JM conducted the study selection. JG and JD piloted the data extraction table. All authors provided critical feedback on the manuscript. All authors reviewed and approved the final manuscript.

## Funding

This work was supported by the Swiss National Science Foundation SNSF through the National Centre of Competence in Research on Robotics, the Vontobel Foundation, the Gemeinnützige Stiftung ACCENTUS, the ETH Zurich Foundation in collaboration with Hocoma AG, and the National Research Foundation, Prime Minister's Office, Singapore under its Campus for Research Excellence and Technological Enterprise (CREATE) program.

## Conflict of Interest

The authors declare that the research was conducted in the absence of any commercial or financial relationships that could be construed as a potential conflict of interest.

## Publisher's Note

All claims expressed in this article are solely those of the authors and do not necessarily represent those of their affiliated organizations, or those of the publisher, the editors and the reviewers. Any product that may be evaluated in this article, or claim that may be made by its manufacturer, is not guaranteed or endorsed by the publisher.

## Abbreviations

ADL: activities of daily living
DOF: degrees of freedom
EEG: electroencephalography
EMG: electromyography
EOG: electrooculography
ERP: event-related potential
FMG: force myography
fMRI: functional magnetic resonance imaging
fNIRS: functional near-infrared spectroscopy
IDS: intention detection strategy
IMU: intertial measurement unit
MEG: magnetoencephalography
MMG: mechanomyography
NASA-TLX: national aeronautics and space administration task load index
QUEST: Quebec user evaluation of satisfaction with assistive technology
SD: standard deviation
SSVEP: steady state visually evoked potential
SUS: system usability scale
ULO: upper-limb orthosis
ULS: upper-limb segment(s)
USE: usefulness, satisfaction ease-of-use.
